# Extending the use of GWAS data by combining data from different genetic platforms

**DOI:** 10.1371/journal.pone.0172082

**Published:** 2017-02-28

**Authors:** E. P. A. van Iperen, G. K. Hovingh, F. W. Asselbergs, A. H. Zwinderman

**Affiliations:** 1 Durrer Center for Cardiovascular Research, Netherlands Heart Institute, Utrecht, The Netherlands; 2 Department of Clinical Epidemiology, Biostatistics and Bioinformatics, Academic Medical Center, Amsterdam, The Netherlands; 3 Department of Vascular Medicine, Academic Medical Center, Amsterdam, The Netherlands; 4 Department of Cardiology, Division Heart & Lungs, UMC Utrecht, The Netherlands; 5 Institute of Cardiovascular Science, Faculty of Population Health Sciences, University College London, London, United Kingdom; University of Texas Health Science Center at San Antonio, UNITED STATES

## Abstract

**Background:**

In the past decade many Genome-wide Association Studies (GWAS) were performed that discovered new associations between single-nucleotide polymorphisms (SNPs) and various phenotypes. Imputation methods are widely used in GWAS. They facilitate the phenotype association with variants that are not directly genotyped. Imputation methods can also be used to combine and analyse data genotyped on different genotyping arrays. In this study we investigated the imputation quality and efficiency of two different approaches of combining GWAS data from different genotyping platforms. We investigated whether combining data from different platforms before the actual imputation performs better than combining the data from different platforms after imputation.

**Methods:**

In total 979 unique individuals from the AMC-PAS cohort were genotyped on 3 different platforms. A total of 706 individuals were genotyped on the MetaboChip, a total of 757 individuals were genotyped on the 50K gene-centric Human CVD BeadChip, and a total of 955 individuals were genotyped on the HumanExome chip. A total of 397 individuals were genotyped on all 3 individual platforms. After pre-imputation quality control (QC), Minimac in combination with MaCH was used for the imputation of all samples with the 1,000 genomes reference panel. All imputed markers with an r^2^ value of <0.3 were excluded in our post-imputation QC.

**Results:**

A total of 397 individuals were genotyped on all three platforms. All three datasets were carefully matched on strand, SNP ID and genomic coordinates. This resulted in a dataset of 979 unique individuals and a total of 258,925 unique markers. A total of 4,117,036 SNPs were available when imputation was performed before merging the three datasets. A total of 3,933,494 SNPs were available when imputation was done on the combined set. Our results suggest that imputation of individual datasets before merging performs slightly better than after combining the different datasets.

**Conclusions:**

Imputation of datasets genotyped by different platforms before merging generates more SNPs than imputation after putting the datasets together.

## Background

In the past decade many Genome-wide Association Studies (GWAS) were executed in order to assess the relative contribution of single-nucleotide polymorphisms (SNPs) in various phenotypes. At this moment more than 21,750 SNPs were significantly associated, in more than 2437 studies (http://www.ebi.ac.uk/GWAS Accessed [May 2016]) with one or more phenotypes by GWAS. These GWAS were performed on a wide range of different genotyping platforms, with a great diversity in the number and density of SNPs, ranging from 50k to 1 million SNPs.

Imputation methods are widely used in GWAS, since this will provide information about variants that are not genotyped directly. Imputation can also be used to combine data genotyped on different genotyping arrays. The quality of imputation is discussed in several papers[1],[2], and imputation methods are used in all large genetic meta-analysis consortia (eg. CARDIoGRAM, MAGIC and GIANT) [3–5]. The standard method used in these large consortia combining data of studies genotyped on different platforms is to impute the individual cohorts locally and combine them later centrally. The most import reason to analyse data using this method is the computational efficiency. Another imputation method mainly used in case-control studies, is to use only SNPs that are common to all the genotyping platform used in the analysis to remove potential platform specific imputation errors, when cases and controls are genotyped on different platforms.

In this paper we investigated whether there is a difference in the imputation quality (number of imputed SNPs with r^2^ > 0.3) and efficiency between imputing different platforms by themselves and combine them afterwards versus combining platforms before the imputation. We hypothesize that combining the individual platforms before imputation will lead to more imputed good quality SNPs. We used the AMC-PAS[6] cohort which is genotyped on 3 different platforms: MetaboChip, 50K gene-centric Human CVD BeadChip and the HumanExome BeadChip.

## Materials and methods

We included a total of 979 unique patients from the previously described prospective cohort AMC-PAS[6], with symptomatic Coronary Artery Disease (CAD) before the age of 51 years, defined as Myocardial Infarction (MI), coronary revascularization, or evidence of at least 70% stenosis in a major epicardial artery. The samples were genotyped on at least one of the three different platforms, all manufactured by Illumina: The MetaboChip[7], The Human cardiovascular disease (HumanCVD) BeadChip (Illumina, San Diego, CA, USA), also known as the ITMAT-Broad-CARe (IBC) (IBCv2 array) [8] and the HumanExome BeadChip(version 24 v1.0) [9]. The MetaboChip consists of approximately 200,000 SNPs chosen based on GWAS meta-analyses of 23 metabolic traits[7].

The 50K gene-centric Human CVD BeadChip has approximately 50,000 SNPs on the array in about 2000 genes in relevant loci across a range of cardiovascular, metabolic and inflammatory syndromes[8].

The HumanExome BeadChip contains about 250,000 variants based on the data of 12,000 sequenced genomes and exomes. Each variant on the chip has been seen at least 3+ times across at least 2 different data sets (http://genome.sph.umich.edu/wiki/Exome_Chip_Design).

A total of 706 individuals were genotyped on the MetaboChip, a total of 757 individuals were genotyped on the 50K gene-centric Human CVD BeadChip and a total of 955 individuals were genotyped on the HumanExome chip. A total of 397 individuals were genotyped on all three platforms. We only included autosomal chromosomes in our analysis.

### Ethics

The Institutional Review Board of the Academic Medical Center approved the protocol. All patients gave written informed consent.

### Pre-imputation quality control

After genotyping, PLINK v1.07 (http://pngu.mgh.harvard.edu/purcell/plink/) was used in the genotype data generated by all 3 platforms to test the SNPs for population substructure which could introduce false-positive associations. This was done by means of multidimensional scaling[10], individuals identified as population outliers were removed from the genotype data from all 3 platforms. Also SNPs were subjected to quality control filters based on sample size and minor allele frequencies (MAF). Samples with a call rate of <95% were excluded from further analysis. Genetic markers with a MAF <1% and Hardy-Weinberg equilibrium p < 10^−4^ were excluded from further analysis. An identity-by-state (IBS) analysis was performed to remove related samples from the analyses. We checked if the genotypes for SNPs available on multiple platforms were concordant, by comparing the genotypes of the SNPs available on all 3 platforms using the–*merge-mode 7* option in PLINK.

### Imputation

Minimac [11] in combination with MaCH[12] were used for the imputation of the combined set of individuals and markers with the 1000 Genomes reference panel (Phase 1 Version march 2012) including all ethnicities. We used a two-step imputation approach. First, the haplotypes of the entire sample were estimated using MaCH followed by haplotype-to-haplotype imputation by Minimac. Minimac generates the allele dosages for each of the variants. Minimac also generates the SNP-level quality metric Rsq (r^2^). The r^2^ value is the SNP-specific estimated squared correlation between the allele dosages and the unknown true genotype. r^2^ is an efficient post-imputation quality control value. We used an r^2^ value of > 0.3 as our post-imputation QC[11,13,14].

To validate our method, we performed the same analysis with the other widely used imputation software IMPUTE2[15] (chromosome 22 only) and we performed the same analysis for only 2 platforms (chromosome 22 only). We imputed all individuals that were genotyped on the Exomechip (N = 853) and the individuals that were only genotyped on the Metabochip (N = 78). First we performed imputation analyses on these two datasets separately, and combined the results afterwards. In our second approach we merged the two data sets before we performed the imputation analyses.

### Association analysis

Finally, we performed association analysis of both imputed data sets for the Low-Density lipoprotein concentration (LDL-C) phenotype relevant for premature cardio atherosclerotic disease. All analyses were adjusted for age and sex. The results were filtered based on bonferroni correction (p-values < 5x10^-8^).

## Results

The number of SNPs available after QC is shown in Table 1. The concordance for SNPs genotyped on all 3 genotyping platforms was perfect. All SNPs genotyped on more than one platform had the same genotype call.

**Table 1:** Number of genotyped SNPs available after QC on the different genotyping platforms.

Array	#Individuals (unique)	#SNPs	Call rate
IBC CardioChip	718	35,092	0.998
MetaboChip	585	113,685	0.997
Exomechip	853	114,967	0.999
Combined	979	258,925	0.739
Combined (only overlapping individuals	397	258,896	0.998455

The call rate of the individual arrays was 99.8%, when we combine the different arrays the call rate drops to 74%, this is because we introduce more missing SNP data, because not all SNPs are available on all 3 platforms.

### Combining different platforms together

**Fig 1:**
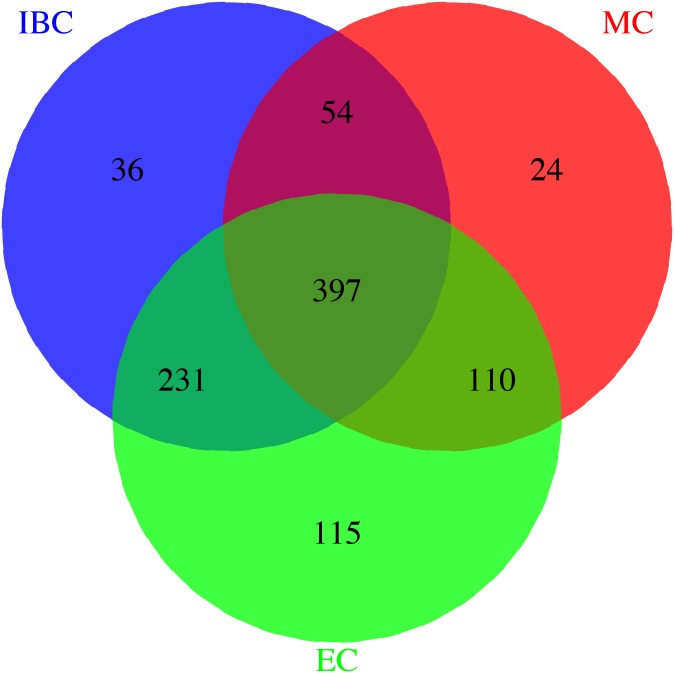
Overlap of individuals genotyped with the three platforms (after QC).

All three datasets were carefully matched on strand, SNP ID and genomic coordinates. This resulted in a dataset of 979 unique individuals and a total of 258,925 SNPs. 397 individuals were genotyped on all three platforms. A total of 36 individuals were only genotyped on the IBC Cardiochip, 24 individuals were only genotyped on the Metabochip and 115 individuals were uniquely genotyped on the Exomechip. The Venn diagram in Fig 1 illustrates the overlap of individuals on the different genotyping platforms after pre-imputation quality control.

### Imputation

The computational time needed to impute a chromosome varied between 153 hours (chr1) and 23 hours(chr22). There was no significant difference between combining the datasets before imputation or combining the datasets after imputation.

After imputation and post-imputation quality control there were a total of 707,871 SNPs available for the IBC Cardiochip array, a total of 2,853,265 SNPs available for the Metabochip, a total of 1,586,399 SNPs for the Exomechip and a total of 3,933,494 SNPs for the combined dataset of those 3 platforms.

**Fig 2:**
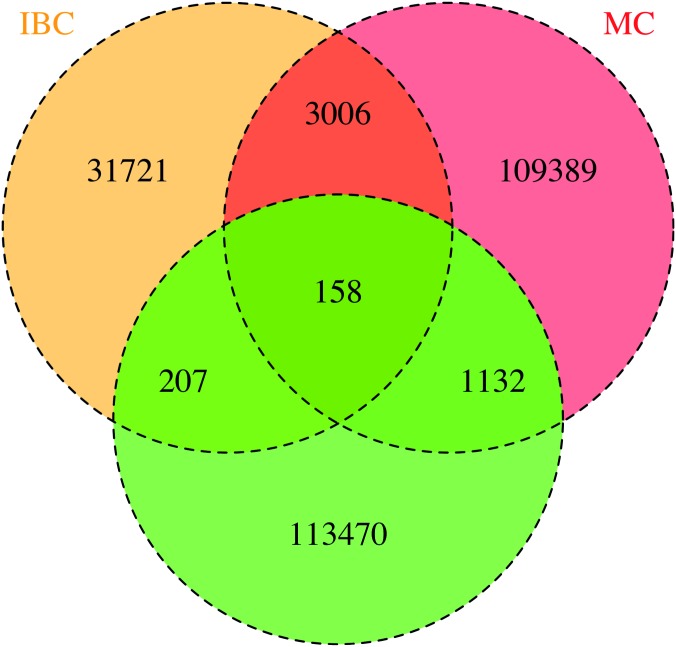
Overlap SNPs after imputation on the different platforms and the combined imputation.

The Venn-diagram in Fig 2 gives an overview of the overlap of the total number of SNPs available after imputation between the different data sets. As an example: 305,373 SNPs were uniquely available after imputation of the MetaboChip. A total of 109,229 unique SNPs were available from each of the 3 different platforms and the combined dataset.

To validate our analyses, we did perform the same analysis with IMPUTE2 for both methods and we did find the same result, combining after imputation leads to more good quality SNPs.

**Fig 3:**
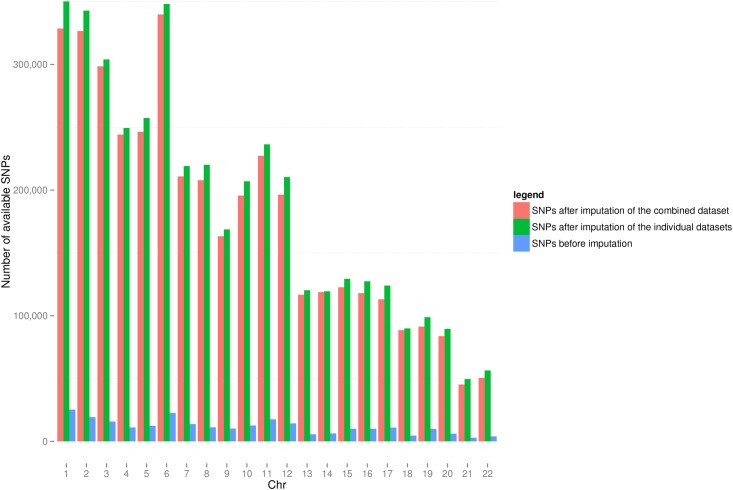
Histogram of number of snps available after both imputation methods.

We observed a difference in the number of available SNPs after imputation between the two imputation approaches. Combining the individual datasets after imputation, resulted in more SNPs than combining the 3 datasets before imputation. A total of 4,117,036 unique SNPs was available after combining the three different sets after separate imputation versus a total of 3,933,494 SNPs after the imputation of the combined set, a 5% difference (ranging between 1% (chr15) and 10% (chr22)) in the number of available SNPs after imputation, see Fig 3.

The overlap of SNPs of the combined data sets and the union of the independently imputed datasets was 3,428,387 SNPs. This meant that 1.242.341 SNPs were only imputed by one of the two different imputation approaches.

**Fig 4:**
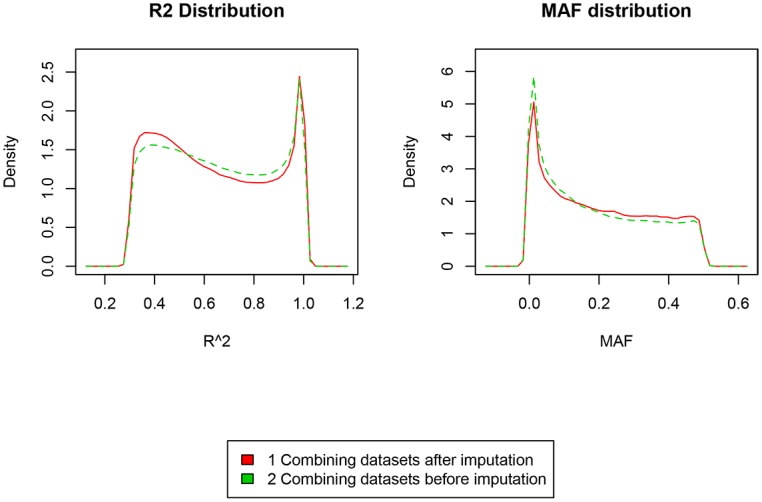
r^2^ and MAF distribution for the two imputation methods.

We also observed that more low frequency variants were imputed with the method when we combined the different data sets after imputation than imputation after combining the datasets, shown in Table 2. Fig 4 shows the r^2^ distribution for all overlapping SNPs for both methods and the MAF distribution for both methods.

**Table 2:** MAF distribution of the two imputation methods.

	#SNPs MAF			
Method	< 1%	> 1% < 5%	>5% <10%	>10%
Combined before imputation	512,579	591,523	486,281	2,343,111
Combined after imputation	615,779	579,945	497,244	2,715,283
% Difference between methods	17%	2%	2%	14%

The analysis where we combined data of 2 platforms after imputation analyses (chromosome 22 only) resulted in a total of 31,241 available SNPs of good quality (r^2^ > 0.3) after merging the 2 seperate imputed datasets. In our second approach we merged the two data sets before we performed imputation analyses, this analyses resulted in a total of 13,516 available SNPs of good quality (r^2^ > 0.3).

We repeated the imputation analysis for the subgroup of individuals that were genotyped on all three platforms (n = 397). The number of SNPs available after combining the three separately imputed sets was 3,245,164 versus a total of 3,528,322 SNPs after the imputation of the combined set, a 8% difference between the 2 imputation methods in favour of the method combining the 3 platforms before imputation.

### Association analysis comparison

**Fig 5:**
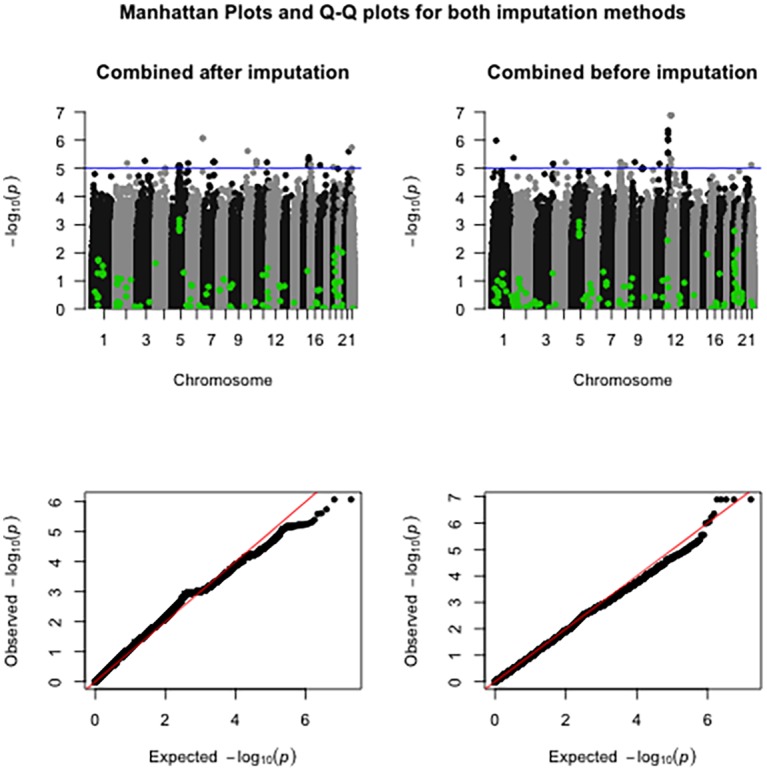
Manhattan plots and Q-Q plots for the association analyses for both imputation methods for LDL-C.

Association analysis was performed in both imputed datasets with the LDL-C phenotype in the AMC-PAS cohort. No significant association was found with the LDL-C phenotype in both methods. Manhattan plots and QQ plots for both imputed datasets are shown in Fig 5.

We have highlighted known associated SNPs reported in the NHGRI GWAS Catalogue (available at: http://www.ebi.ac.uk/gwas/) for the LDL cholesterol trait. After removing duplicate SNPs, a total of 118 SNPs were available. Of these, 113 SNPs were available in the dataset combined before imputation analyses and 105 SNPs in the dataset combined after imputation. None of the p-values of these known LDL-C associated SNPs was associated with LDL-c in our analyses. In both datasets a variant in the HMGCR gene was the most significant known LDL-c associated SNP. In the dataset combined after imputation rs3846662 p = 6.62x10^-4^ and in the dataset combined before imputation rs7703051 p = 7.97x10^-4^.

## Discussion

Combining different imputed GWAS datasets is a very powerful method to identify new loci using data from different genotyping platforms[16].

Our study illustrates that combining imputed data from different platforms before and after imputation does result in differential numbers and quality of SNPs to be analysed. When imputation of different data sets is performed prior to combining resulted in 5% more SNPs that satisfied the post-imputation QC, this was not what we hypothesized and we deem those to explained by the r^2^ selection during the imputation process. When a SNP is genotyped on only one of the platforms, al lot more uncertainty will be introduced in the set when we combined the 3 platforms before imputation, because this SNP is only available on one of the three platforms. When we apply a call-rate filter of 95% or higher, we found the opposite result, the method where we combine the datasets before imputation results in more good quality SNPs. To validate our method, we performed the same analysis with IMPUTE2[15] (chromosome 22) and we found the same result, combined after imputation leads to more good quality SNPs. To validate our chosen r^2^ threshold we did run the analysis with different r^2^ thresholds (r^2^ > 0.5 and r^2^ > 0.8), resulting in the same difference of good quality SNPs between the different imputation methods.

We found the opposite result when we analysed only data from individuals (N = 397) genotyped on all 3 platforms, where we were able to impute 8% more SNPs that satisfied our post-imputation QC when we first combine before performing imputation. We think this is because we do not introduce extra missing data.

To validate our findings we performed the same analysis for only 2 platforms (chromosome 22 only). This result is in favour of combining after imputation and is in line with our previous results.

Imputation of the individual datasets seems to result in more good quality SNPs (r^2^ > 0.3). However, several limitations apply to our study. Firstly, the results presented here were based on 3 different gene-centric genotyping platforms. This meant that the variant density around known and interesting genes is higher than on a normal GWAS platforms. Therefore, the results of our analyses can be different if applied to non-gene-centric GWAS datasets. Secondly, the relatively small sample size of 979 unique individuals in de AMC-PAS cohort could have influenced the total number of variants with good quality available after the imputation. Thirdly, due to the relative small sample size we did not find a significant difference in computational time needed for both imputation methods. In datasets with more individuals and SNPs, the computation time needed for the combined imputation method will increase exponentially, this is the main reason why the combined after imputation is seen as the golden-standard within all large consortia at the moment.

On the other hand, we have a unique data collection, there are few cohorts genotyped on 2 or more different platforms. Other studies have analysed the quality of imputed SNPs after combining the genotyping data of different cohorts on different genotyping platforms[17].

## Conclusion

In conclusion, our results indicate that combining the data from three different platforms together after imputation performs better than combining the data of the 3 platforms before imputation.

## Consent

All patients gave informed consent.

## Abbreviations

CAD: Coronary Artery Disease
GWAS: Genome-wide Association Studies
IBS: identity by state
LDL-C: Low-density lipoprotein concentration
MAF: Minor Allele Frequency
MI: Myocardial Infarction
QC: Quality Control
SNPs: single-nucleotide polymorphisms
